# Vitamin D Deficiency Aggravates the Renal Features of Moderate Chronic Kidney Disease in 5/6 Nephrectomized Rats

**DOI:** 10.3389/fmed.2018.00282

**Published:** 2018-10-10

**Authors:** Ana Carolina de Bragança, Daniele Canale, Janaína Garcia Gonçalves, Maria Heloisa Massola Shimizu, Antonio Carlos Seguro, Rildo Aparecido Volpini

**Affiliations:** ^1^Laboratorio de Investigacao Medica 12 (LIM12), Hospital das Clinicas HCFMUSP, Faculdade de Medicina, Universidade de São Paulo, São Paulo, Brazil; ^2^Laboratorio de Investigacao Medica 12 (LIM12), Faculdade de Medicina, Universidade de São Paulo, São Paulo, Brazil

**Keywords:** chronic kidney disease, vitamin D deficiency, fibrosis, inflammation, 5/6 nephrectomy, experimental model

## Abstract

The pathogenesis of chronic kidney disease (CKD) involves a very complex interaction between hemodynamic and inflammatory processes, leading to glomerular/vascular sclerosis, and fibrosis formation with subsequent evolution to end-stage of renal disease. Despite efforts to minimize the progression of CKD, its incidence and prevalence continue to increase. Besides cardiovascular diseases and infections, several studies demonstrate that vitamin D status could be considered as a non-traditional risk factor for the progression of CKD. Therefore, we investigated the effects of vitamin D deficiency (VDD) in the course of moderate CKD in 5/6 nephrectomized rats (Nx). Adult male Wistar rats underwent Sham surgery or Nx and were subdivided into the following four groups: Sham, receiving standard diet (Sham); Sham VDD, receiving vitamin D-free diet (VDD); Nx, receiving standard diet (Nx); and VDD+Nx, receiving vitamin D-free diet (VDD+Nx). Sham or Nx surgeries were performed 30 days after standard or vitamin D-free diets administration. After validation of vitamin D depletion, we considered only Nx and VDD+Nx groups for the following studies. Sixty days after surgeries, VDD+Nx rats exhibited hypertension, a greater decline in renal function and plasma FGF-23 levels, renal hypertrophy, as well as higher plasma levels of PTH and aldosterone. In addition, those animals presented more significant chronic tubulointerstitial changes (cortical interstitial expansion/inflammation/fibrosis), higher expression of collagen IV, fibronectin and α-smooth muscle actin, and lower expressions of JG12 and M2 macrophages. Also, VDD+Nx rats had greater infiltration of inflammatory cells (M1 macrophages and T-cells). Such changes were accompanied by higher expression of TGF-β1 and angiotensinogen and decreased expression of VDR and Klotho protein. Our observations indicate that vitamin D deficiency impairs the renal function and worsens the renovascular and morphological changes, aggravating the features of moderate CKD in 5/6 nephrectomized rats.

## Introduction

The incidence and prevalence of chronic kidney disease (CKD) have been increasing over the years (1). The pathogenesis of CKD involves a complex interaction between hemodynamic and inflammatory processes, which involves glomerular/vascular sclerosis and fibrosis formation. Subsequently, the set of those features evolves to end-stage renal disease (ESRD) (1). It has been shown that the mortality of patients with CKD is directly related to renal function deterioration associated with cardiovascular/hemodynamic diseases and infections (2, 3). These major risk factors account for only half of the causes of mortality (2, 3). Therefore, early intervention against risk factors during the course of CKD is crucial to avoid the progression of the disease.

Lately, several lines of evidence have been highlighting the vitamin D [25(OH)D] status and its role as a non-traditional risk factor for renal diseases (2, 3). The kidneys play an important role in the metabolism of vitamin D, which in turn, is responsible for renal protection and regulation of many physiological activities. Besides osteogenesis and maintenance of mineral homeostasis (4, 5), vitamin D also controls a multitude of additional biological activities linked to the immune and cardiovascular systems, skin and muscle function, cellular growth control, and numerous additional biological processes (6). Vitamin D synthesis is a well described process that begins in the skin and continues in the liver and the kidney, the main site of production of the active form of vitamin D 1,25(OH)_2_D_3_ or calcitriol (4, 5, 7, 8). 25(OH)D circulates in the blood bound to the vitamin D-binding protein (DBP) and the uptake of the complex 25(OH)D-DBP from the glomerular ultrafiltrate is mediated by the transmembrane protein megalin (7, 8). Once inside proximal tubular cells, 25(OH)D is either delivered to renal 1α-hydroxylase for its conversion to 1,25(OH)_2_D_3_ to maintain its own production as well as renal and endocrine vitamin D receptor (VDR) activation (7, 8).

Renal diseases, even in the early stages, are usually accompanied by decreased levels of 25(OH)D and 1,25(OH)_2_D_3_ (7, 8). Thus, low levels of vitamin D can be deleterious and impair the recovery of renal diseases and/or even accelerate the progression of renal disease. In a previous study, we showed that vitamin D deficiency exerted a pivotal role on the aggravation of acute kidney injury (AKI) after ischemia/reperfusion (I/R) insult (9). We also demonstrated that vitamin D deficiency favored renal capillary rarefaction, and increased renal fibrosis and inflammation (10). Furthermore, we monitored the evolution of ischemic AKI and observed important chronic tubulointerstitial changes were aggravated by vitamin D deficiency (2).

As mentioned above, evidence suggest that renal diseases are generally followed by low levels of vitamin D. However, there is a lack of studies on vitamin D deficiency on the different stages of CKD. Notably, 5/6 nephrectomy (Nx) is a classical experimental model that mimics CKD in humans (1, 11). Histological studies in the remnant kidney after renal ablation have shown a complex response identified by three phases: (1) a hypertrophic phase, comprising glomerular hypertrophy and tubular dilatation; (2) a quiescent phase, with minimal histological alterations; and (3) an end stage, with segmental and focal glomerulosclerosis development and tubulointerstitial fibrosis (12, 13). The third and last phase of Nx model encompasses the features of intermediate stages of CKD to ESRD, characterized by progressive and irreversible decline in renal function associated with histomorphological changes (14).

Therefore, in light of the aforementioned evidence that renal diseases are associated with low levels of vitamin D, our aim was to investigate the influence of vitamin D deficiency on renal changes in a moderate stage of CKD in 5/6 nephrectomized rats.

## Materials and methods

### Experimental protocol

Forty adult male Wistar rats (*Rattus novergicus*), weighing 180-200 g, were used in this study. The rats were obtained from a local facility at the Faculty of Medicine-University of Sao Paulo. The experimental procedures were specifically approved by the local Research Ethics Committee (CEUA, registration 122/16) and developed in strict accordance with our institutional guidelines and with well-established international standards for the care and use of laboratory animals. All surgeries were performed under appropriate anesthesia, and all efforts were made to minimize suffering. During the 90-day experiment, all animals were maintained under standard laboratory conditions, receiving vitamin D-free or standard diets (MP Biomedicals, Irvine, CA) and free access to tap water.

At first, in order to evaluate several biochemical parameters (Table 1), we subdivided the animals into 4 groups: (Sham) Sham-operated rats (*n* = 10) received a standard diet for 90 days; (VDD) Vitamin D deficiency (*n* = 10), received a vitamin D-free diet for 90 days and also submitted to sham surgery; (Nx) 5/6 nephrectomy (*n* = 10), received a standard diet for 90 days and submitted to renal ablation; and (VDD+Nx) Vitamin D deficiency plus 5/6 nephrectomy (*n* = 10), received a vitamin D-free diet for 90 days and submitted to renal ablation as well. On day 30, 5/6 nephrectomy (Nx) was performed in a single-step procedure (Nx and VDD+Nx groups). A suprapubic incision was performed under anesthesia with 2,2,2-Tribromoethanol [250 mg/Kg body weight (BW)]. After that, the right kidney was removed, and two or three branches of the left renal artery were ligated, resulting in the infarction of two-thirds of the left kidney and the incision was sutured immediately. Also on day 30, sham-operated rats (Sham and VDD groups), underwent anesthesia and manipulation of the renal pedicles without any reduction of renal mass. All groups were followed for another 60 days after the surgeries. Thus, this subdivision of the animals into Sham, VDD, Nx, and VDD+Nx groups was planned to verify whether the vitamin D-free diet induced the depletion of this hormone in VDD and VDD+Nx groups. Once we confirmed the efficiency of the vitamin D depletion model, we considered only Nx and VDD+Nx groups for the following studies. By that, we kept the focus of this work on investigating the influence of vitamin D deficiency in Nx experimental model.

**Table 1:** Renal function, hemodynamic and biochemical parameters after 90 days evaluated in Sham rats, Vitamin D deficient rats (VDD), rats submitted to 5/6 nephrectomy (Nx), and Vitamin D deficient rats submitted to 5/6 nephrectomy (VDD+Nx).

	**Sham**	**VDD**	**Nx**	**VDD+Nx**
BW (g)	437.3 ± 19.5	409.3 ± 9.8	438.5 ± 12.4	417.0 ± 15.8
GFR (mL/min/100g BW)	0.65 ± 0.01	0.59 ± 0.03	0.48 ± 0.03^a^^f^	0.39 ± 0.03^a^^d^^i^
MAP (mmHg)	115.0 ± 5.7	135.9 ± 4.2^c^	147.6 ± 6.0^b^	167.1 ± 5.2^a^^e^^i^
P_Ur_ (mg/dL)	34.95 ± 1.03	41.17 ± 3.41	89.89 ± 7.76	109.7 ± 27.60^c^^f^
P_Ca_ (mg/dL)	8.15 ± 0.40	7.63 ± 0.07	8.15 ± 0.015	7.50 ± 0.14
P_P_ (mg/dL)	4.52 ± 0.37	4.70 ± 0.25	4.58 ± 0.15	4.17 ± 0.15
25(OH)D (ng/mL)	49.49 ± 2.87	<0.44^a^ undetectable	42.26 ± 3.97^f^	<0.44^a^^g^ undetectable
PTH (pg/mL)	311.2 ± 44.2	899.8 ± 167.6	1023.0 ± 334.3	901.7 ± 184.3
FGF-23 (pg/mL)	424.0 ± 56.6	327.5 ± 37.8	648.7 ± 44.2^b^^d^	484.6 ± 44.5^h^

*BW, body weight; GFR, inulin clearance; MAP, mean arterial pressure; P_Ur_, plasma urea concentration; P_Ca_, plasma calcium concentration; P_P_, plasma phosphorus concentration; 25(OH)D, 25 hydroxyvitamin D; PTH, parathormone; FGF-23, fibroblast growth factor 23. Values are mean ± SEM*.

a *p < 0.001*,

b *p < 0.01*,

c *p < 0.05 vs. Sham*;

d *p < 0.001*,

e *p < 0.01*,

f *p < 0.05 vs. VDD*;

g *p < 0.001*,

h *p < 0.01*,

i *p < 0.05 vs. Nx*.

### Inulin clearance and hemodynamic studies

On day 90, the animals were anesthetized with sodium thiopental (50 mg/Kg BW) and placed on a temperature-regulated surgical table. The trachea was cannulated (PE-240 catheter) and spontaneous breathing was maintained. The jugular vein was cannulated (PE-60 catheter) for infusion of inulin and fluids. To monitor mean arterial pressure (MAP) and collect blood samples, the right carotid artery was catheterized with a PE-50 catheter. We assessed MAP with a data acquisition system (MP100; Biopac Systems, Santa Barbara, CA). To collect urine samples, the urinary bladder was cannulated (PE-240 catheter) by suprapubic incision. After completion of the cannulation surgical procedure, a loading dose of inulin (100 mg/Kg BW diluted in 1 mL of 0.9% saline) was administered through the jugular vein. Subsequently, a constant infusion of inulin (10 mg/kg BW in 0.9% saline) was started and continued at 0.04 mL/min throughout the whole experiment. Three urine samples were collected at 30-min intervals. Blood samples were obtained at the beginning and at the end of the experiment. Inulin clearance values represent the mean of three periods. Blood and urine inulin were determined by the anthrone method, and the glomerular filtration rate (GFR) data is expressed as ml/min/100g BW.

### Biochemical parameters

To assess plasma levels of 25-hydroxyvitamin D [25(OH)D], parathormone (PTH), fibroblast growth factor 23 (FGF-23), aldosterone, phosphate (P_P_) and calcium (P_Ca_), we collected blood samples after the clearance studies. We assessed 25(OH)D, PTH, FGF-23 and aldosterone by enzyme-linked immunosorbent (ELISA) using commercial kits: 25-Hydroxyvitamin D (ALPCO, Salem, NH); Rat Intact PTH and Mouse/Rat Intact FGF-23 (Immutopics, Inc., San Clemente, CA); and Aldosterone (Enzo Life Sciences, Farmingdale, NY). P_P_ and P_Ca_ were evaluated by colorimetric assay (Labtest, Lagoa Santa/MG, Brazil).

### Tissue sample preparation

After the clearance experiment, we perfused kidneys with phosphate-buffered solution (PBS, pH 7.4). Fragments of right kidneys were frozen in liquid nitrogen and stored at−80°C for western blotting and real-time quantitative polymerase chain reaction (qRT-PCR). Fragments of left kidneys were fixed in methacarn solution (60% methanol, 30% chloroform, 10% glacial acetic acid) for 24 h and in 70% alcohol thereafter. Kidney blocks were embedded in paraffin and cut into 4-μm sections for histology and immunohistochemistry.

### Total protein isolation

Kidney samples were homogenized in ice-cold isolation solution (200 mM mannitol, 80 mM HEPES and 41 mM KOH, pH 7.5) containing a protease inhibitor cocktail (Sigma Chemical Company, St. Louis, MO) in a homogenizer (PT 10/35; Brinkmann Instruments, Westbury, NY). Homogenates were centrifuged at 4000 x rpm for 30 min at 4°C to remove nuclei and cell debris. Supernatants were isolated, and protein was quantified by Bradford assay (Bio-Rad Laboratories, Hercules, CA).

### Western blot assays

For western blot analysis, 100 μg of total kidney protein were separated on SDS-polyacrylamide minigels by electrophoresis (15). After transfer by electroelution to PVDF membranes (GE Healthcare Limited, Little Chalfont, UK), blots were blocked for 1 h with 5% nonfat dry milk in Tris-buffered saline solution. Blots were then incubated overnight with primary antibodies for: TGF-β1 (1/500; Santa Cruz Biotechnology, Santa Cruz, CA); angiotensinogen (AGT) (1/200; Santa Cruz Biotechnology); α-Klotho (1/500; Santa Cruz Biotechnology); and VDR (1/500; Santa Cruz Biotechnology). The labeling was visualized with horseradish peroxidase-conjugated secondary antibody (anti-rabbit or anti-mouse IgG, 1:2000, or anti-goat, 1:10000; Sigma Chemical, St. Louis, MO) and enhanced chemiluminescence (ECL) detection (GE Healthcare Limited, Little Chalfont, UK). Kidney protein levels were further analyzed with a gel documentation system (Alliance 4.2; Uvitec, Cambridge, UK) and the software Image J for *Windows* (Image J–NIH Image). We used densitometry to quantitatively analyze the protein levels, normalizing the bands to actin expression (anti β-actin, Sigma Chemical, St. Louis, MO).

### Light microscopy

Four-micrometer histological sections of kidney tissue were stained with Masson's trichrome and examined under light microscopy. We quantified the fractional interstitial area (FIA) by analyzing tubulointerstitial involvement and glomerular tuft area as well. For histomorphometry, the images obtained by microscopy were captured on a computer screen via an image analyzer software (ZEN, Carl Zeiss, Munich, Germany). For FIA evaluation, we analyzed 30–40 grid fields (0.09 mm^2^ each) per kidney cortex. The interstitial areas were demarcated manually, and the proportion of the field was determined after excluding the glomeruli. For glomerular area, we calculated the arithmetic mean after analyzing approximately 80 glomeruli of each kidney section. The glomerular tuft area (μm^2^) was circulated manually and calculated automatically by the software. To minimize bias in the morphometric analysis, the observer was blinded to the treatment groups.

### Immunohistochemical analysis

Immunohistochemistry was performed on 4-μm-thick paraffinized kidney sections mounted on 2% silane-coated glass slides. We used the following antibodies: (1:100) mouse monoclonal to CD68 (ED1; BioRad, Hercules, CA); (1:2000) rabbit polyclonal to mannose receptor (CD206; Abcam, Cambridge, MA); (1:50) mouse monoclonal to CD3 (DAKO, Glostrup, Denmark); (1:200) mouse monoclonal to α-smooth muscle actin (α-SMA) (Millipore, Billerica, MA); (1:200) rabbit polyclonal to collagen IV (Abcam, Cambridge, MA); (1:500) rabbit monoclonal to fibronectin (Abcam, Cambridge, MA); (1:150) rabbit polyclonal to vitamin D receptor (VDR; Santa Cruz Biotechnology, Santa Cruz, CA); and (1:150) mouse monoclonal to JG12, direct against to aminopeptidase P (Santa Cruz Biotechnology, Santa Cruz, CA). We subjected the kidney tissue sections to immunohistochemical (IHC) reaction according to the protocol for each primary antibody. Reaction products were detected by avidin-biotin-peroxidase (Vector Laboratories, Burlingame, CA). The color reaction was developed in 3,3-diaminobenzidine (Sigma, St. Louis, MO) and hydrogen peroxide. Counterstaining was with Harris' hematoxylin. We analyzed 40-60 renal cortex fields (0.09 mm^2^) to evaluate the results of immunoreactions. For that, the volume ratios of positive areas of renal tissue (%), determined by the color limit, were obtained by an image analyzer software (ZEN, Carl Zeiss, Munich, Germany) on a computer coupled to a microscope (Axioskop 40; Carl Zeiss) and a digital camera (2, 16). To minimize bias in the IHC analysis, the observer was blinded to the treatment groups.

### Gene expression

We performed real-time qPCR in frozen renal tissue, assessing the following genes: *VDR* (Rn00690616_m1) and *Klotho* (Rn00580123_m1). We extracted and prepared total RNA by centrifugation technique using the commercial kit *SV Total RNA Isolation System* (Promega Corporation, WI). For cDNA synthesis, we used total RNA and GoTaq qPCR master mix reagent (Promega, Madison, WI). We performed real-time PCR using TaqMan (Applied Biosystems, Foster City, CA) on Step One Plus (Applied Biosystems). Primers were purchased from Applied Biosystems. We evaluated relative gene expression with the 2^−ΔΔ*Ct*^ method (17), using glyceraldehyde 3-phosphate dehydrogenase (*GAPDH*) as the housekeeping gene (Rn01775763_g1).

### Statistical analysis

All quantitative data were expressed as mean ± SEM (standard error of the mean). Differences among groups were analyzed with GraphPad Prism 5.0 software (GraphPad Software, La Jolla, CA) by one-way analysis of variance followed by the Student-Newman-Keuls test. Comparisons between groups were made by unpaired *t*-test. Values of *p* < 0.05 were considered statistically significant.

## Results

### Body weight, renal function and mean arterial blood pressure

As described in Table 1, there were no differences in BW among the groups. We estimated the food intake over the experimental protocol (90 days) and all animals showed similar food ingestion (~25 g/day). Our inulin clearance studies (mL/min/100g BW) showed that renal ablation led to an impaired renal function, evidenced by GFR compared to Sham rats. VDD+Nx rats presented a significantly greater loss of GFR (Table 1). Corroborating our renal function data, plasma urea concentration (mg/dL) was higher in VDD+Nx rats than in Sham and VDD rats (Table 1). In addition, we also found a higher MAP (mmHg) in those animals from VDD and Nx groups compared to Sham (Table 1). VDD+Nx rats showed higher levels of MAP (Table 1). Therefore, our GFR and MAP results indicate that vitamin D deficiency is an aggravating factor concerning renal function recovery and blood pressure control.

### Vitamin D, PTH, and other biochemical parameters

As previously described, our animals were maintained on a standard or a vitamin D-free diets for 90 days. Predictably, over the experimental period, the plasma levels of 25(OH)D (ng/mL) from VDD and VDD+Nx rats were undetectable (<0.44; Table 1). Regarding plasma PTH levels (pg/mL), we did not find any difference among the experimental groups (Table 1). Also reported in Table 1, the evaluation of plasma FGF-23 (pg/mL) showed higher levels of this hormone in Nx rats compared to Sham and VDD rats. On the other hand, vitamin D deficiency significantly decreased the plasma concentration of FGF-23, as observed in VDD+Nx rats (Table 1). Plasma levels of phosphorus and calcium did not change among the experimental groups (Table 1). The implementation of vitamin D deficiency model was confirmed through hemodynamics and biochemical parameters (Table 1). Henceforward, we focused our study directly on the model of renal ablation under vitamin D deficiency, considering only Nx and VDD+Nx groups.

### Vitamin D deficiency impairs blood pressure control

**Figure 1:**
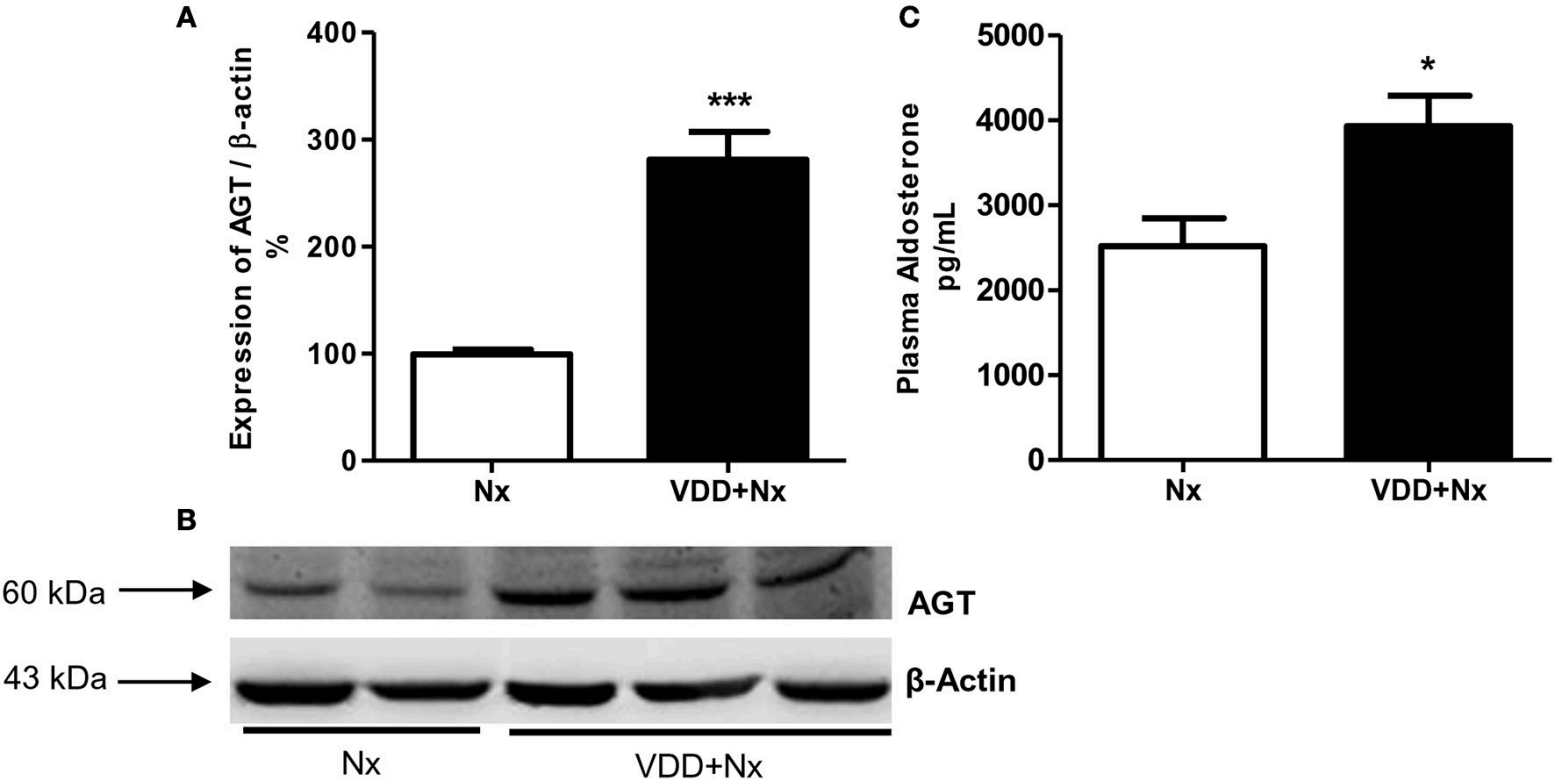
Semiquantitative immunoblotting for angiotensinogen (AGT) expression in rat kidney tissue and plasma aldosterone levels. **(A)** Densitometric analysis of samples from Nx and VDD+Nx rats. **(B)** Representative immunoblots which reacted with anti-AGT revealing a 60 kDa band. **(C)** Plasma aldosterone levels. Data are mean ± SEM. *p < 0.05 and ***p < 0.001 vs. Nx. (Nx, 5/6 nephrectomy; VDD+Nx, vitamin D deficiency + 5/6 nephrectomy).

In order to further investigate the role of vitamin D on blood pressure control, we evaluated the protein expression of AGT and plasma aldosterone levels as well. AGT protein expression (%) was significantly higher in VDD+Nx rats compared to Nx rats (Figure 1). In addition, we found higher levels of aldosterone (pg/mL) in VDD+Nx rats compared to Nx rats (Figure 1). Taken together, our results are consistent with a role of vitamin D on blood pressure control.

### Vitamin D deficiency and Klotho—FGF-23 interaction

**Figure 2:**
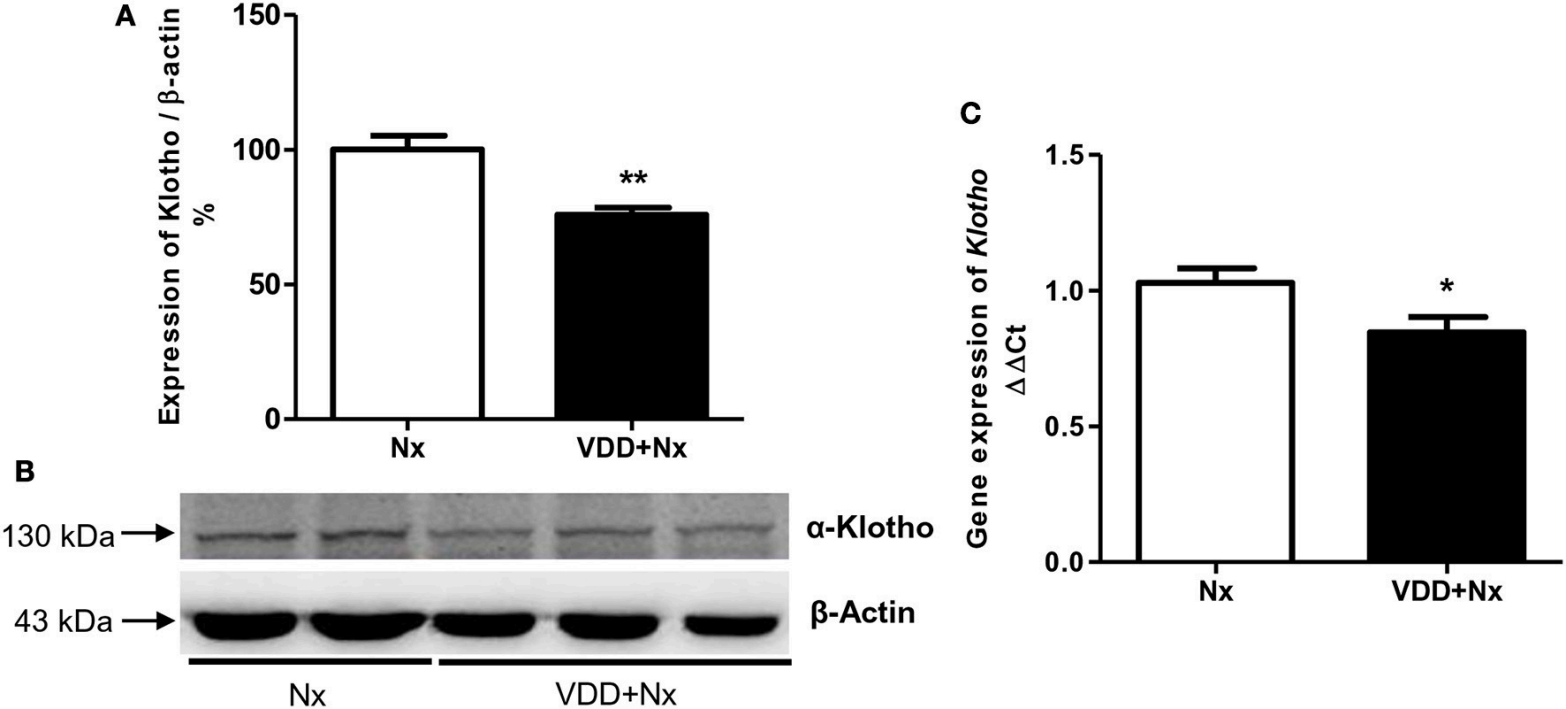
Semiquantitative immunoblotting and real-time quantitative polymerase chain reaction analysis for Klotho expression in rat kidney tissue. **(A)** Densitometric analysis of samples from Nx and VDD+Nx rats. **(B)** Representative immunoblots which reacted with anti-Klotho revealing a 130 kDa band. **(C)** Bar graph of *Klotho* gene expression values. Data are mean ± SEM. *p < 0.05 and **p < 0.01vs. Nx. (Nx, 5/6 nephrectomy; VDD+Nx, vitamin D deficiency + 5/6 nephrectomy).

Klotho increases the affinity of specific fibroblast growth factor receptor (FGFR) isoforms for FGF-23 binding. Both Klotho and FGF-23 have been emerging as early markers of the different stages of CKD (18). In addition to our FGF-23 data described above (Table 1), we also evaluated both Klotho protein (%) and gene expression (ΔΔCt) in renal tissue samples. We observed that vitamin D deficiency decreased Klotho protein expression in VDD+Nx rats in comparison to Nx rats. In addition, vitamin D deficiency reduced *Klotho* gene expression in VDD+Nx rats compared to Nx rats (Figure 2). Thus, collectively our results highlight the intrinsic role of vitamin D concerning its interaction with Klotho and FGF-23 on the kidney-parathyroid endocrine axis.

### Vitamin D deficiency exacerbates histomorphological changes

**Figure 3:**
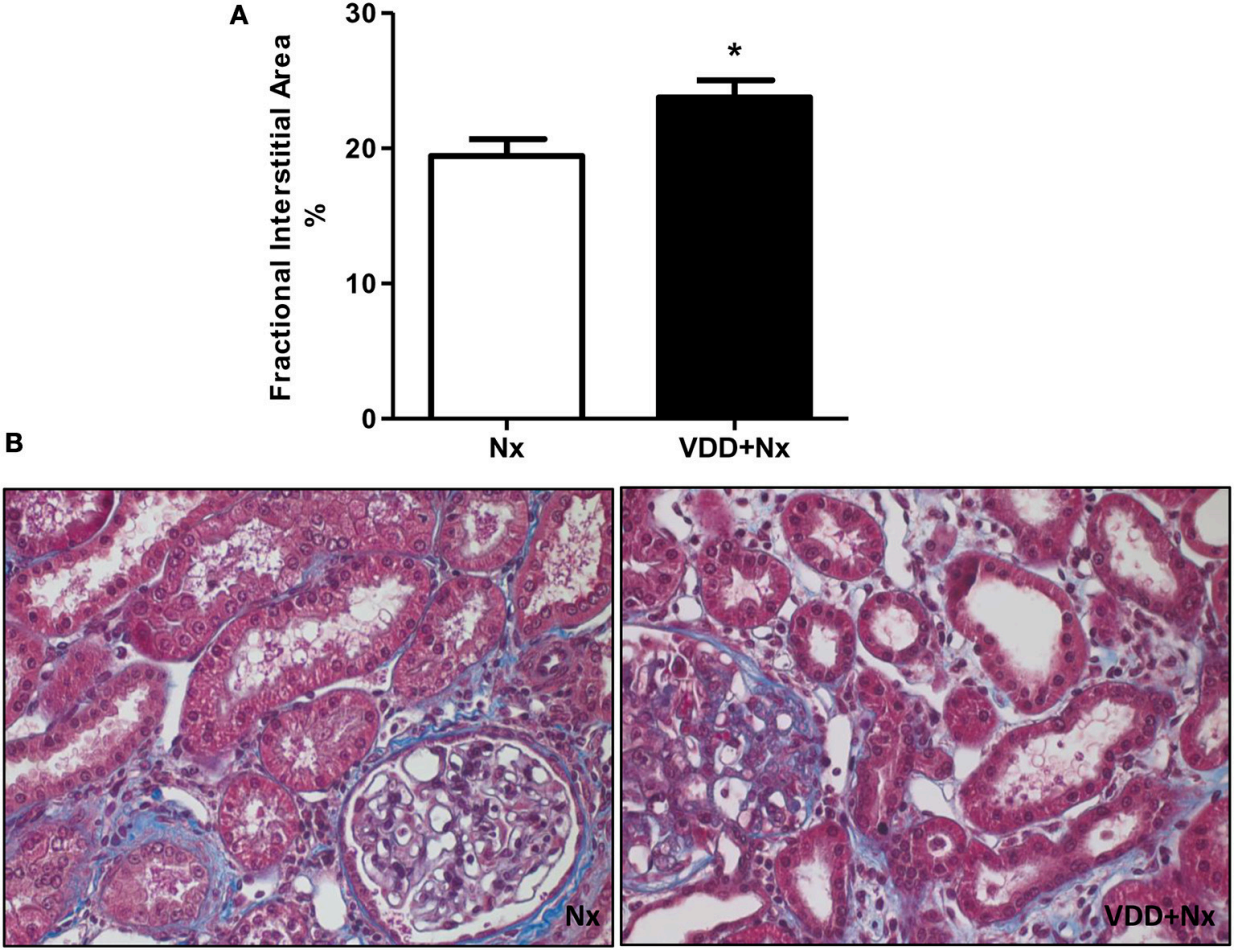
Fractional interstitial area in rat kidney tissue. **(A)** Bar graph of fractional interstitial area values. **(B)** Representative photomicrographs of renal histological changes in a Nx rat and in a VDD+Nx rat (x400). Data are mean ± SEM. *p < 0.05 vs. Nx. (Nx, 5/6 nephrectomy; VDD+Nx, vitamin D deficiency + 5/6 nephrectomy).

Nx is a well-known experimental model that features CKD, which includes the presence of segmental and focal glomerulosclerosis and tubulointerstitial fibrosis (12, 13). Our light microscopy studies revealed histological alterations such as interstitial fibrosis, tubular atrophy and dilatation, and inflammatory cell infiltrates in the renal cortex of Nx and VDD+Nx rats (Figure 3). In addition, morphometric studies were performed to evaluate the FIA of the renal cortex. We observed evident alterations in the tubulointerstitial compartment, featuring interstitial expansion (renal fibrosis and inflammatory cell infiltrates) in both Nx and VDD+Nx rats. Figure 3 displays that FIA (%) was significantly greater in VDD+Nx rats than in Nx rats, showing that vitamin D deficiency exerts an important role on interstitial morphological alterations. We did not find any difference in glomerular tuft area (μm^2^) between two groups (Nx: 11,502 ± 507 vs. VDD+Nx: 11,198 ± 450).

### Vitamin D deficiency aggravates renal inflammation

**Figure 4:**
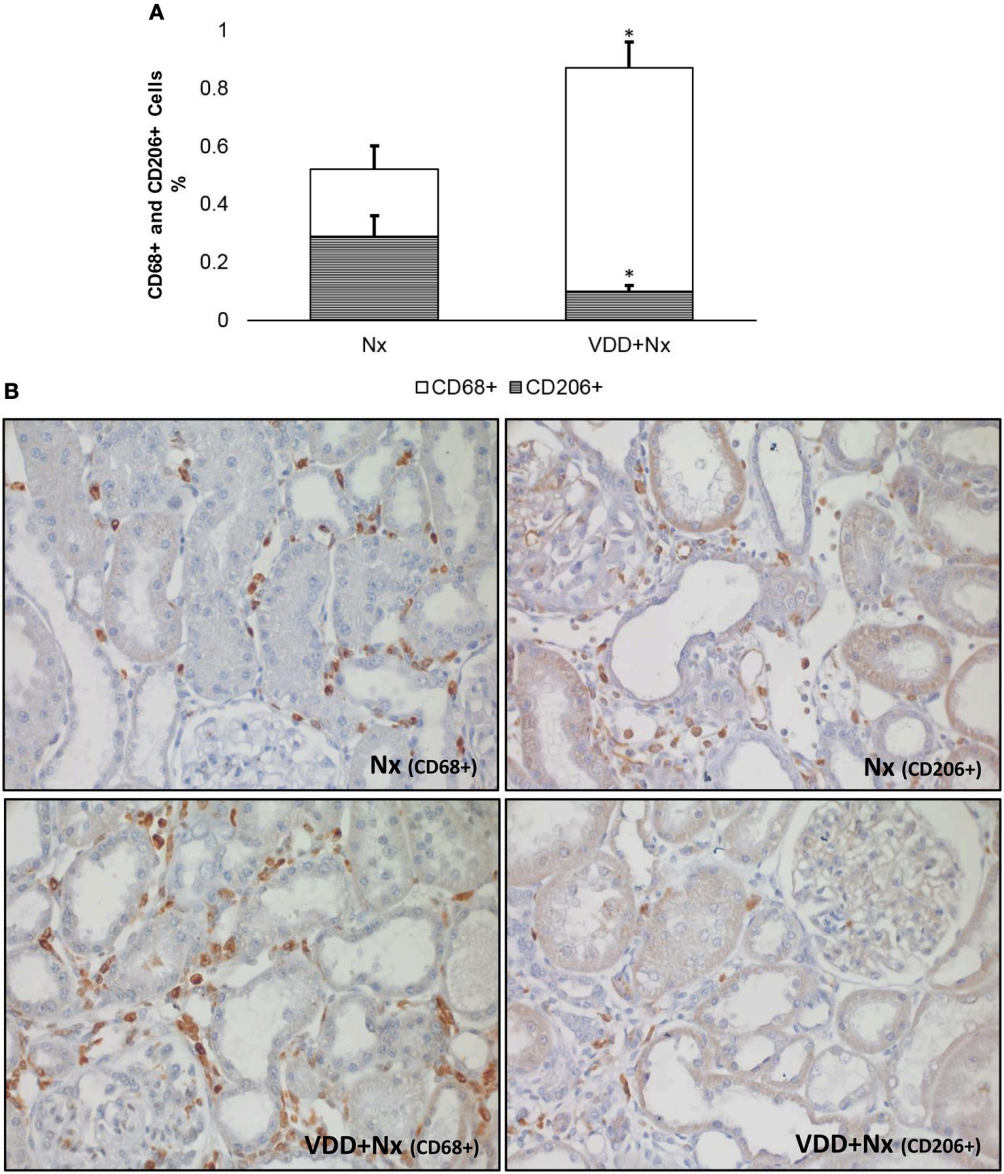
Immunohistochemical analysis of CD68+ cells (M1+M2 macrophages) and CD206+ cells (M2 macrophages) expression in rat kidney tissue. **(A)** Bar graph of the proportion of CD206+ cells in relation to the amount of CD68+ cells. **(B)** Representative photomicrographs of immunostaining for CD68+ and CD206+ cells in the renal cortex of a Nx rat and a VDD+Nx rat (x400). Note that vitamin D deficiency not only increased the expression of CD68+ cells but also reduced the expression of CD206+ cells. Data are mean ± SEM. *p < 0.05 vs. Nx. (Nx, 5/6 nephrectomy; VDD+Nx, vitamin D deficiency + 5/6 nephrectomy).

**Figure 5:**
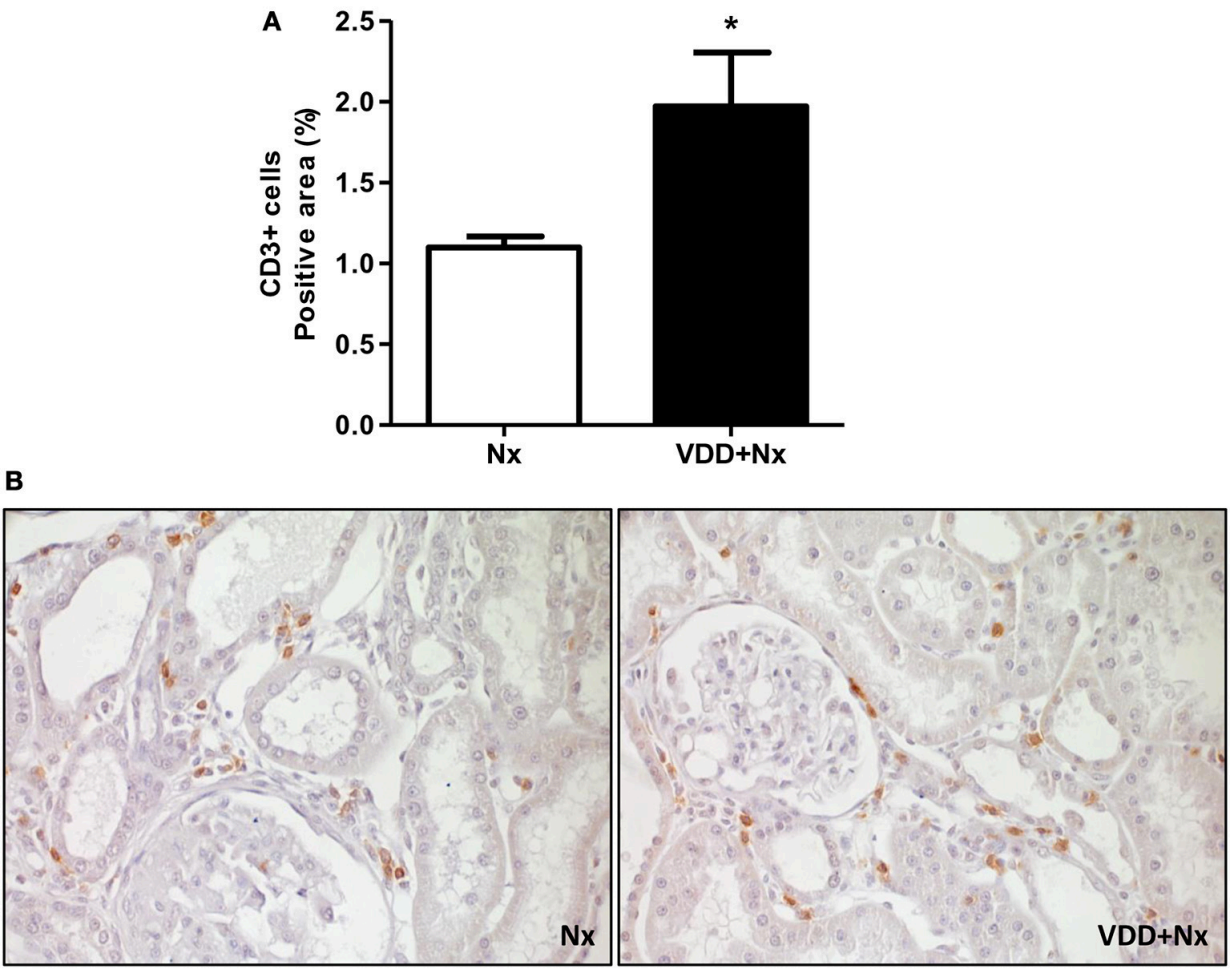
Immunohistochemical analysis of CD3+ cells (T-cells) expression in rat kidney tissue. **(A)** Bar graph of CD3+ cells expression values. **(B)** Representative photomicrographs of immunostaining for CD3+ cells in the renal cortex of a Nx rat and a VDD+Nx rat (x400). Data are mean ± SEM. *p < 0.05 vs. Nx. (Nx, 5/6 nephrectomy; VDD+Nx, vitamin D deficiency + 5/6 nephrectomy).

As described above, our histological studies revealed the presence of inflammatory cell infiltration in the renal cortex of Nx and VDD+Nx groups. Thus, we assessed the renal expression of CD68 (macrophages) and CD3 (T-cells) positive cells by IHC studies. As shown in Figure 4, the percentage of tubulointerstitial cells stained for CD68 (%) was significantly higher in VDD+Nx rats than in Nx rats. Likewise, Figure 5 shows that the percentage of tubulointerstitial cells staining for CD3 (%) was significantly higher in VDD+Nx rats than in Nx rats. Therefore, our results reinforce the important role of vitamin D on the modulation of the immune/inflammation system.

### Vitamin D deficiency impairs renal tissue repair

In response to tissue injury, macrophages become activated based on specific signals from the damaged microenvironment. In our study the macrophage population was immunolocalized by an anti-CD68 antibody, also known as ED1, and included both M1 and M2 macrophages. M1 macrophages are considered as proinflammatory cells whereas type M2 are macrophages related to tissue repair (19, 20). As state above and represented in Figure 4, we showed an increased percentage of CD68+ cells (M1+M2 macrophages) mainly in VDD+Nx group. Next, we investigated the population of macrophages stained by CD68 performing IHC studies for CD206, also known as mannose receptor, which is an exclusive marker for M2 macrophages. As shown in Figure 4, the percentage of CD206+ cells (%) was significantly lower in VDD+Nx rats than in Nx rats. For a better overview, we calculated the proportion of M2 macrophages (CD206+ cells) in relation to the general population of macrophages (M1+M2) evaluated by CD68. Nx rats presented a lower percentage of CD68+ cells and a higher proportion of CD206+ cells. Meanwhile, VDD+Nx rats showed a much lower proportion of CD206+ cells based on the amount of CD68+ cells found in the kidney of these animals (Figure 4). Hence, we could infer that vitamin D deficiency not only caused a larger infiltration of inflammatory cells but also impaired the tissue repair provided by M2 macrophages.

### Vitamin D deficiency, secretion of ECM components and phenotypic alteration

**Figure 6:**
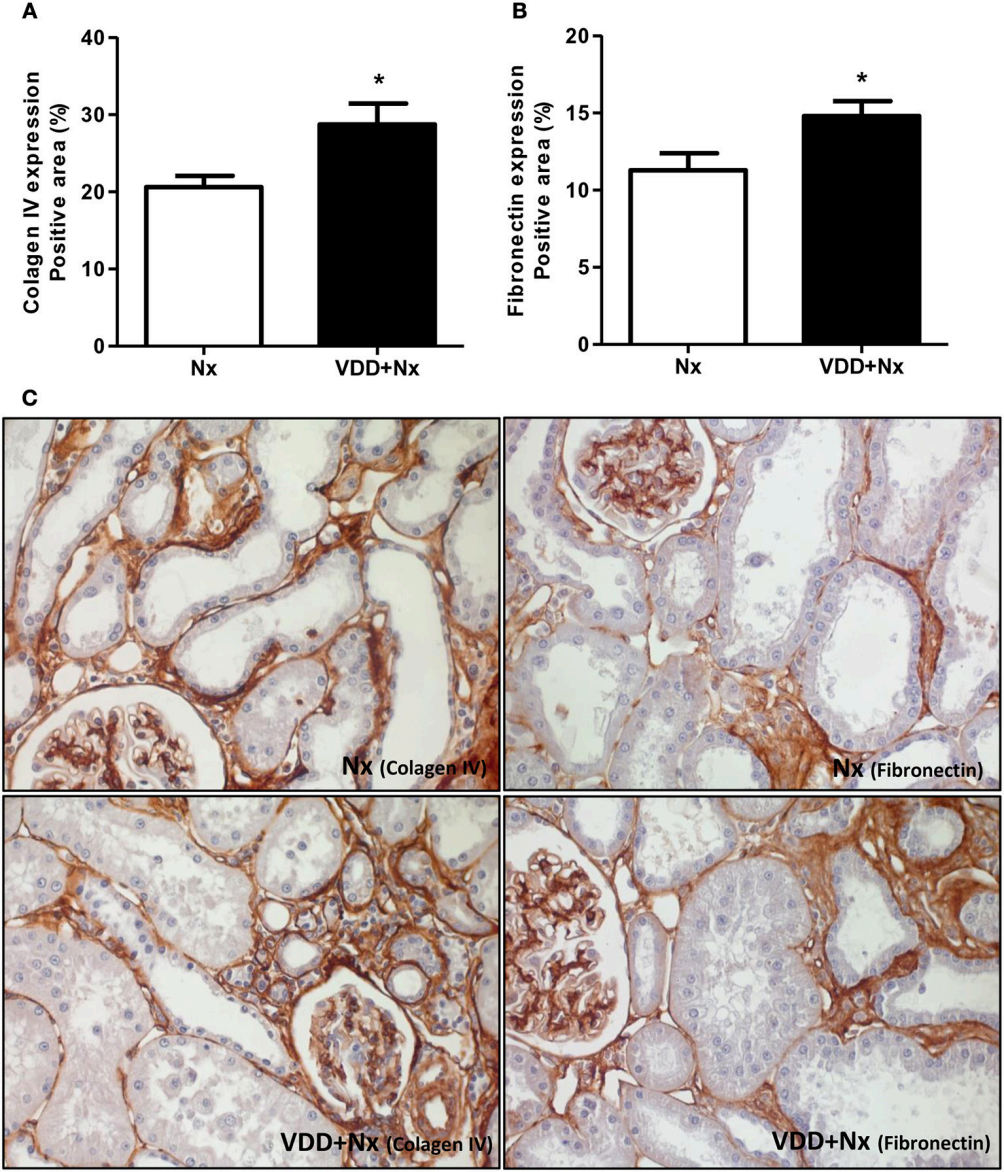
Immunohistochemical analysis of collagen IV and fibronectin expression in rat kidney tissue. Bar graph of **(A)** collagen IV and **(B)** fibronectin expression values. **(C)** Representative photomicrographs of immunostaining for collagen IV and fibronectin in the renal cortex of a Nx rat and a VDD+Nx rat (x400). Data are mean ± SEM. *p < 0.05 vs. Nx. (Nx, 5/6 nephrectomy; VDD+Nx, vitamin D deficiency + 5/6 nephrectomy).

**Figure 7:**
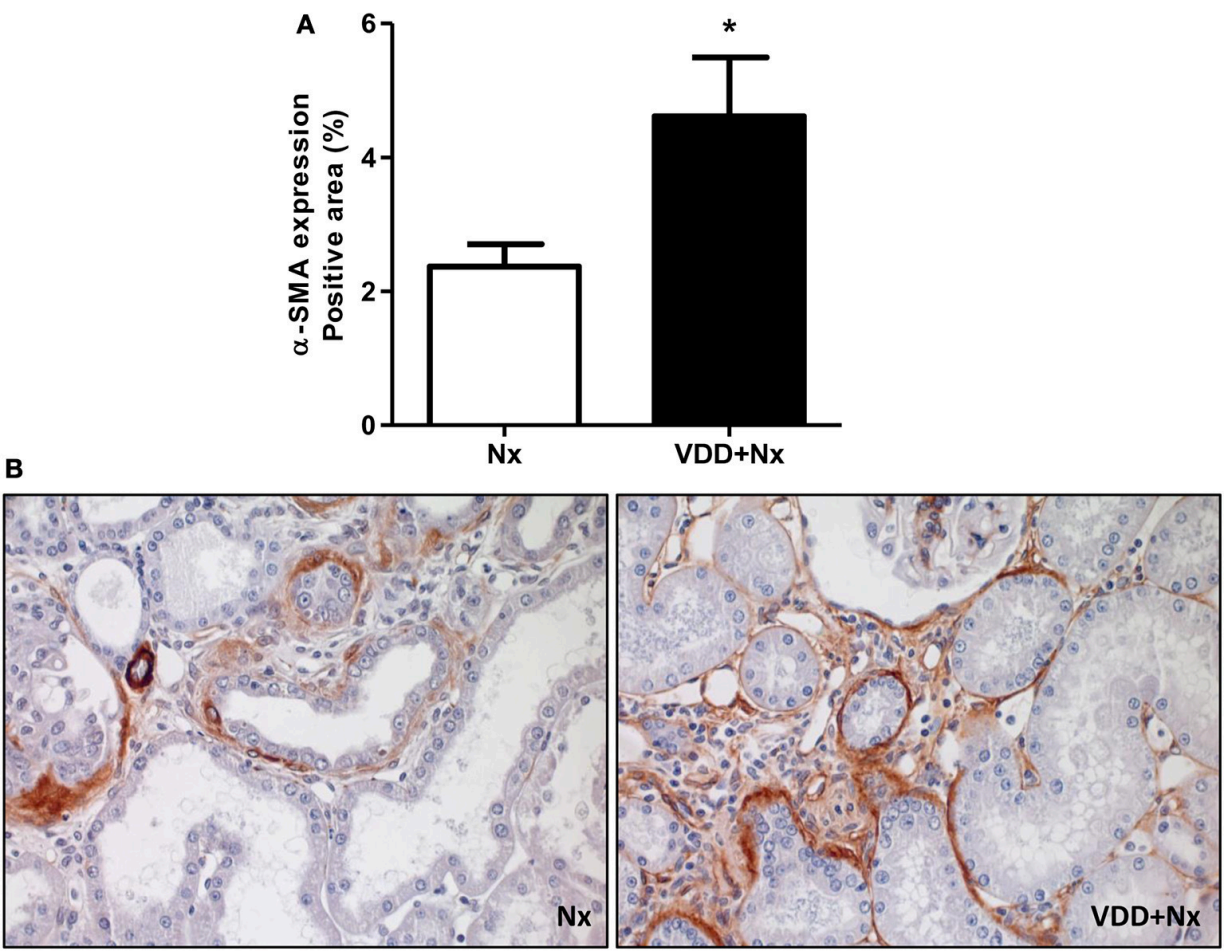
Immunohistochemical analysis of α-smooth muscle actin (α-SMA) expression in rat kidney tissue. **(A)** Bar graph of α-SMA expression values. **(B)** Representative photomicrographs of immunostaining for α-SMA in the renal cortex of a Nx rat and in VDD+Nx rat (x400). Data are mean ± SEM. *p < 0.05 vs. Nx. (Nx, 5/6 nephrectomy; VDD+Nx, vitamin D deficiency + 5/6 nephrectomy).

As mentioned before, our histomorphological data showed that vitamin D deficiency aggravated the expansion of tubulointerstitial compartment and fibrosis formation. This process is complex and involves the production and secretion of many extracellular matrix (ECM) components. Thus, we performed IHC studies for type IV collagen and fibronectin, two fibrous components of ECM. Our data, represented as percentage of positive area (%), showed a higher expression of type IV collagen and fibronectin in the renal cortex from VDD+Nx rats compared to Nx rats (Figure 6). Besides ECM markers, we also aimed to evaluate the presence of phenotypic alteration of renal tubular cells. For that, we evaluated the expression of α-SMA as a marker for interstitial fibroblast activation. As illustrated in Figure 7, the percentage of positive area for α-SMA (%) was higher in VDD+Nx rats than in Nx rats. Vitamin D deficiency enhanced the immunostainings for both ECM markers as well as for α-SMA.

### Vitamin D deficiency, VDR expression and renal fibrosis

**Figure 8:**
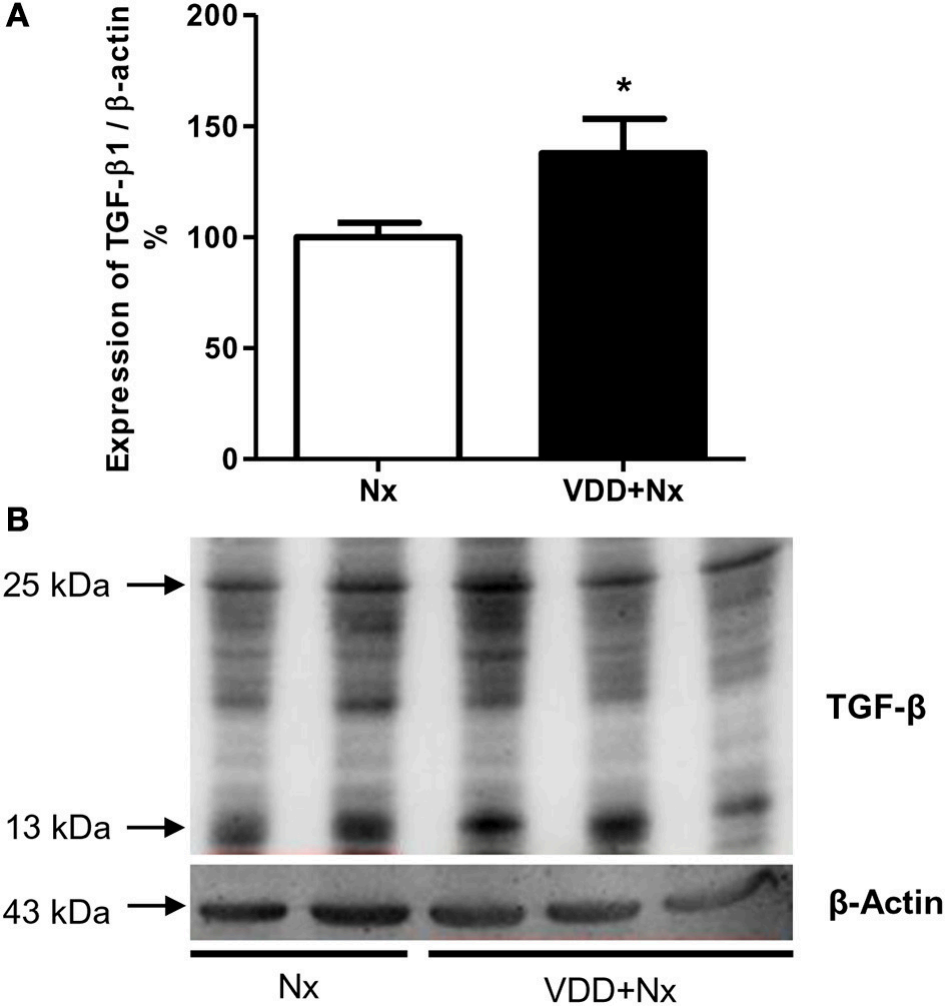
Semiquantitative immunoblotting for transforming growth factor-β1 (TGF-β1) expression in rat kidney tissue. **(A)** Densitometric analysis of samples from Nx and VDD+Nx rats. **(B)** Representative immunoblots which reacted with anti-TGF-β1 revealing both 13 and 25 kDa bands. Data are mean ± SEM. *p < 0.05 vs. Nx. (Nx, 5/6 nephrectomy; VDD+Nx, vitamin D deficiency + 5/6 nephrectomy).

**Figure 9:**
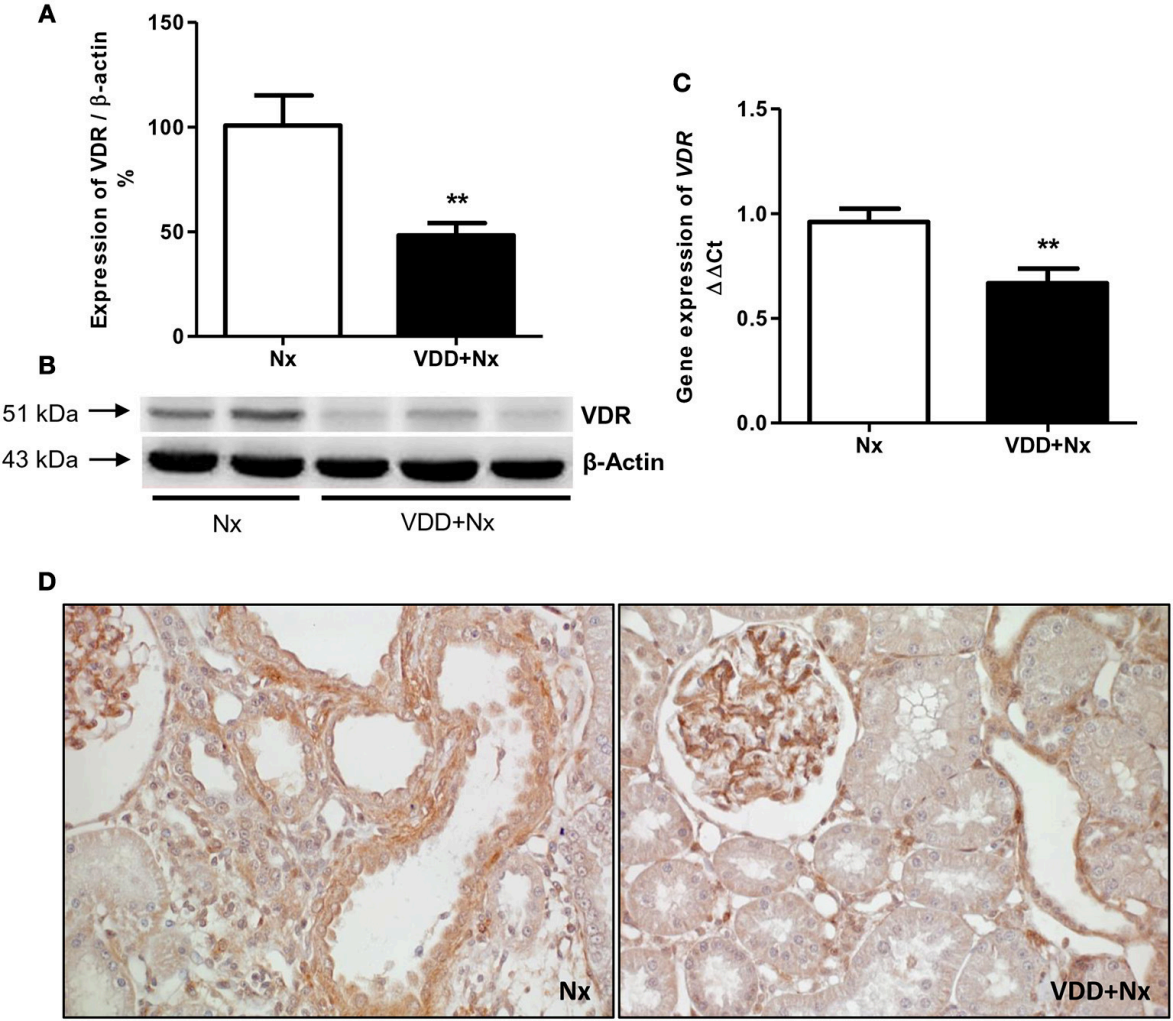
Semiquantitative immunoblotting, real-time quantitative polymerase chain reaction analysis and immunohistochemical study for vitamin D receptor (VDR) expression in rat kidney tissue. **(A)** Densitometric analysis of samples from Nx and VDD+Nx rats. **(B)** Representative immunoblots which reacted with anti-VDR revealing a 51 kDa band. **(C)** Bar graph of *VDR* gene expression values. **(D)** Representative photomicrographs of immunostaining for VDR in the renal cortex of a Nx rat and a VDD+Nx rat (x400). Data are mean ± SEM. **p < 0.01 vs. Nx. (Nx, 5/6 nephrectomy; VDD+Nx, vitamin D deficiency + 5/6 nephrectomy).

Our histological and IHC studies revealed a potential involvement of vitamin D deficiency with renal fibrosis exacerbation. To assess this possible link, we investigated the relationship between TGF-β with VDR expression. As shown in Figure 8, TGF-β1 protein expression was significantly higher in VDD+Nx rats than in Nx rats. Concerning VDR evaluation, we observed a lower gene expression of this receptor in VDD+Nx rats than in Nx rats (Figure 9). Moreover, VDR protein expression and IHC staining was significantly lower in VDD+Nx rats than in Nx rats (Figure 9). Thus, vitamin D deficiency predictably caused a decrease in VDR expression and this alteration influenced the TGF-β1 expression in VDD+Nx rats. Of note, this set of data indicated a closely connection between VDR expression and the complex process of renal fibrosis formation.

### Vitamin D deficiency and glomerular vascular endothelium

**Figure 10:**
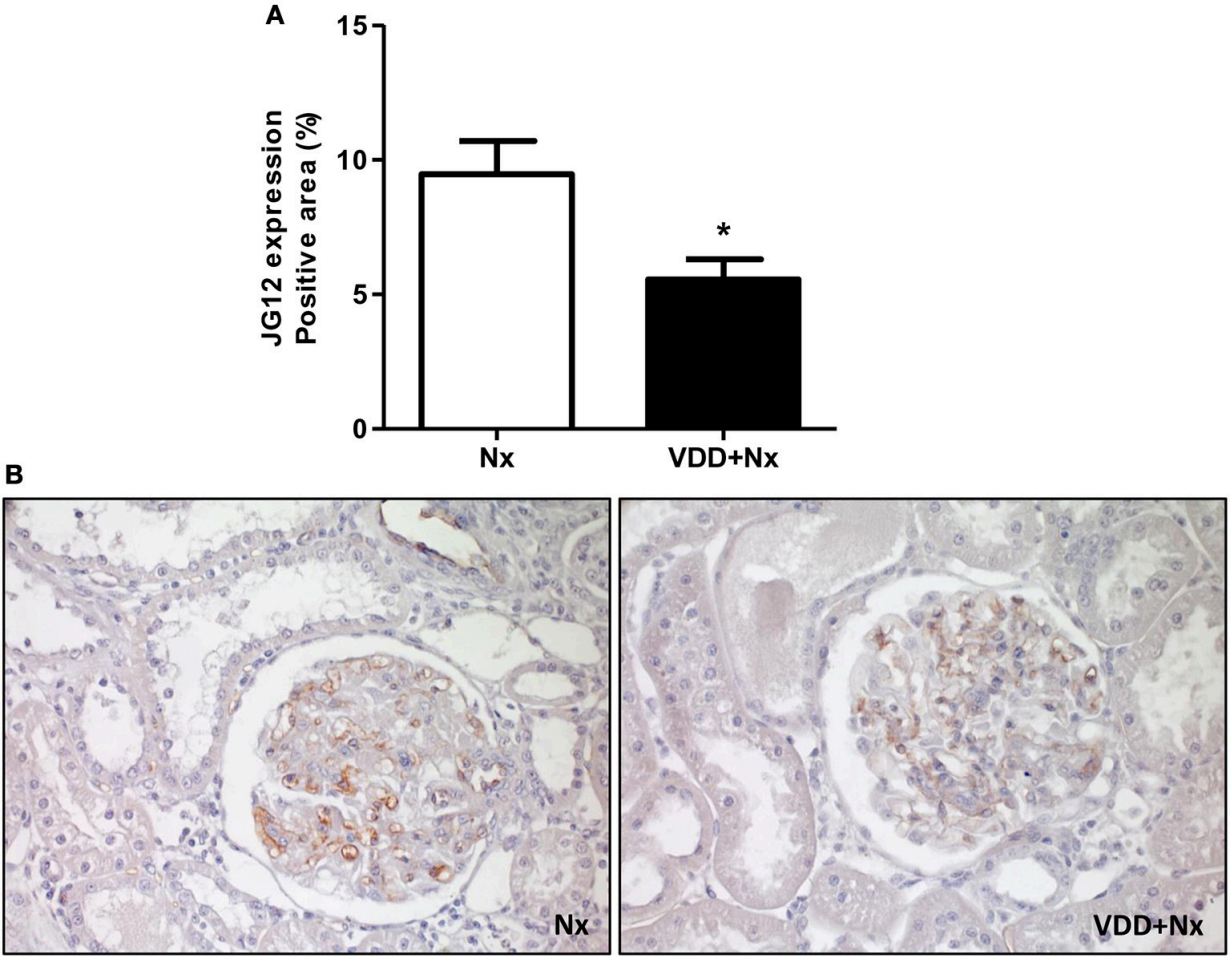
Immunohistochemical analysis of aminopeptidase P (JG12) expression in rat kidney tissue. **(A)** Bar graph of JG12 expression values. **(B)** Representative photomicrographs of immunostaining for JG12 in the renal cortex of a Nx rat and a VDD+Nx rat (x400). Data are mean ± SEM. *p < 0.05 vs. Nx. (Nx, 5/6 nephrectomy; VDD+Nx, vitamin D deficiency + 5/6 nephrectomy).

The damage caused in the renal vascular endothelium is one of the main factors involved in the pathological alterations present in CKD. In our study, we performed IHC studies to evaluate the expression of JG12 as a specific marker for the vascular endothelium. Within the glomerular capsule, JG12 is only expressed on the surface of the capillary endothelium. As shown in Figure 10, JG12 IHC staining per glomerular tuft area (%) was significantly lower in VDD+Nx rats than in Nx rats. Thus, vitamin D deficiency may have impaired the integrity of glomerular vascular endothelium of VDD+Nx rats.

## Discussion

Vitamin D deficiency is present in a considerable number of critical patients and is associated with increased mortality among such patients. Clinical studies have identified low serum vitamin D level as a risk factor for both AKI in critically ill patients and progression to CKD (2, 10, 21, 22). Here, we showed that vitamin D deficiency aggravated the features of moderate CKD induced by 5/6 nephrectomy. We found a significant decline in GFR and increased levels of blood pressure and plasma aldosterone aggravated by vitamin D deficiency. Furthermore, we observed an enlargement of the FIA (fibrosis and inflammatory cells) in VDD+Nx rats. Vitamin D deficiency also contributed to a higher expression of ECM components and to phenotypic modification of renal tubular cells as well. On the other hand, tissue repair and renal vascular endothelium were compromised by vitamin D deficiency. All those changes were accompanied by higher expression of TGF-β1, lower expression of VDR and Klotho protein and decreased plasma FGF-23 levels in VDD+Nx rats.

Our results clearly show that animals fed the vitamin D-free diet presented undetectable levels of 25(OH)D. Humans acquire the majority of their vitamin D from sunlight-induced cutaneous synthesis (approximately 80%), the rest comes from diet and supplements (2, 23). There is a common understanding that low serum 25(OH)D levels cause a negative calcium balance, secondary hyperparathyroidism, and bone disease. However, we did not find significant differences in calcium, phosphorus and PTH serum levels between Nx and VDD+Nx rats, indicating that our animals were in moderate stage of CKD.

Five-sixths renal ablation is a model of CKD accompanied by impaired renal function and, depending on the type of subtotal nephrectomy, by systemic hypertension (24). In this study, Nx and VDD+Nx rats presented a significant decrease in inulin clearance consistent with a moderate stage of CKD concerning loss of renal function. Notably, vitamin D deficiency caused a remarkable decline in GFR compared to Nx rats, reinforcing that vitamin D status is an important factor for renal function preservation in CKD. The pathogenesis of CKD involves a complex interaction between hemodynamic and inflammatory processes (1). In addition to cardiovascular and hemodynamic diseases, hypovitaminosis D has been emerging as a non-traditional risk factor for CKD (2, 3, 25). In 2011, de Boer et al. suggested that low serum vitamin D level is a risk factor for CKD progression, showing that vitamin D supplementation slowed that progression (22). Corroborating this evidence, recent studies also confirmed that the use of vitamin D metabolites and analogs in the treatment of CKD was effective in terms of slowing the risk of progression to ESRD (25, 26).

We also investigated the role of vitamin D on blood pressure control. Our rats submitted to 5/6 nephrectomy presented high levels of blood pressure and this alteration was more severe in VDD+Nx rats. Moreover, this change was accompanied by an increased protein expression of AGT and high levels of plasma aldosterone in VDD+Nx rats. There is strong evidence from studies conducted in humans and animals showing that vitamin D status can modulate the renin-angiotensin-aldosterone system (RAAS) activity, mainly by lowering renin synthesis (25, 27–29). Also, it has been demonstrated that vitamin D deficiency can lead to an upregulation of the RAAS activity, changes in the endothelium, and vascular smooth cells as well (29–31). Supporting our data, we found similar results concerning blood pressure control in a model of CKD progression after I/R. More significantly, we showed that vitamin D deficiency itself induced hypertension in control rats and aggravated the hypertension in rats under hypovitaminosis D euthanized 60 days after I/R (2). Schwarz et al. using a similar procedure, induced 5/6 nephrectomy by surgical resection (avoiding hyperreninism) instead of vascular ligation, with no significant effect of calcitriol treatment on blood pressure in a model of relatively modest renal damage (24).

Regardless of the underlying disease, CKD can be viewed as a process of progressive loss of functional nephrons (32, 33). Despite substantial loss of nephrons, hyperphosphatemia is observed only in late stages of ESRD (CKD stage 4-5) (32, 33). As described above, we did not find any difference in plasma levels of phosphate in our animals, corroborating that our rats were in a moderate stage of CKD. The maintenance of phosphate homeostasis, despite the loss of nephrons, is compensated by increasing the phosphate excretion, which is attained by increase in FGF-23. In this study, we observed higher levels of FGF-23 in Nx rats whereas VDD+Nx rats did not presented this similar profile. In addition to promote renal phosphate excretion, FGF-23 suppresses the production of vitamin D by inhibition of 1-α-hydroxylase and stimulation of 24-hydroxylase (2, 32–37). Moreover, some findings have supported an increasingly and important role of FGF-23 as an initial event in the development of CKD. The first step for that is featured by increased levels of FGF-23 preceding changes in calcium, phosphorus, PTH, or even vitamin D levels, that is, its respective regulatory factors (2, 25, 32, 33, 38, 39). In our study we found lower levels of FGF-23 in VDD+Nx rats, similar to our previous results in a model of CKD progression (2). This low FGF-23 levels could be acting as a compensatory response to prevent further reductions in vitamin D levels, which could exacerbate the forthcoming hypocalcemia in the late stages of CKD (2, 40).

An intrinsic interaction between FGF-23 and Klotho has been described (32, 33). *Klotho* is a putative aging suppressor gene encoding a single-pass transmembrane co-receptor that makes the FGFR specific for FGF-23 (18). In CKD, as the renal disease advances, a decline in Klotho expression has been reported whereas FGF-23 expression increases progressively; high serum phosphate and PTH and low vitamin D levels accompany these changes (18, 32, 33, 41). In this study, both Western blotting and qPCR experiments showed a significant reduction in Klotho expression in VDD+Nx rats associated with low levels of FGF-23 compared to Nx rats. In a previous study of CKD progression, we showed that vitamin D deficiency caused a reduction in Klotho expression in rats euthanized 60 days after I/R injury. More important, our data also demonstrated that vitamin D alone decreased Klotho expression in control rats (2). Clinical data concerning CKD show that changes in FGF-23, Klotho, PTH, and vitamin D are detectable in early CKD, with the exception of serum phosphate which rises later in more advanced CKD (32, 33). Thus, our data confirmed that even in moderate stage of CKD there are evident disturbances of the Klotho-FGF-23 axis. More importantly, vitamin D deficiency contributed to remarkable changes in this sensitive endocrine axis.

The diagnosis of CKD rests on establishing a chronic reduction in kidney function and structural kidney damage (26). As expected, 5/6 nephrectomy resulted in renal morphological changes such as necrosis, cast formation, tubular collapse and dilatation, inflammatory infiltration cells and fibrosis. Associated with these alterations, we found an enlargement of the tubulointerstitial compartment due to the presence of infiltration of inflammatory cells and fibrosis, mainly in the renal cortex of VDD+Nx rats. Corroborating our data, Gonçalves et al. also observed an involvement of the tubulointerstitial compartment characterized by increased FIA as a result of renal fibrosis and inflammatory infiltrate in rats euthanized 60 days after I/R injury. Furthermore, the authors showed that vitamin D deficiency *per se* or associated with I/R injury exacerbated the renal morphological alterations (2).

The activation of tubular epithelial cells results in the secretion of proinflammatory and profibrotic mediators that promote interstitial inflammation and fibrosis (14, 42). In order to go further, we investigated the expression of two fibrous ECM components (fibronectin and type IV collagen) and infiltrating CD68+ and CD3+ cells (macrophages and T-cells, respectively). Vitamin D deficiency enhanced the expressions of fibronectin, collagen IV, CD68+ and CD3+ cells in the renal cortex of VDD+Nx rats. Thus, these data support our results concerning the increased FIA found in VDD+Nx rats. It is important to emphasize that, although the kidney is the main site of production of calcitriol, many different cell types, including immune and inflammatory cells, can locally produce calcitriol (43). During the course of CKD, progressive reductions in GFR and also in renal megalin expression aggravate the low renal uptake of 25(OH)D caused by vitamin D deficiency/insufficiency (7, 8). Depending on the stage, CKD can reduce not only renal 1α-hydroxylase and the amount of filtered 25(OH)D but also renal megalin content and the uptake of 25(OH)D by monocytes–macrophages, markedly impairing both endocrine and autocrine VDR activation (7, 8).

The accumulating ECM in the scarred kidney includes both increased amounts of normal and abnormal renal types of ECM (42). In addition to vascular damage and the activation of inflammatory responses, the factor of initial injury to renal cell can also lead to the fibrotic process, including pro-fibrogenic cytokine releasing such as TGF-β by macrophages and apoptotic parenchymal cells and activation of collagen-producing cells, among others (2, 44). During kidney injury, a major source of TGF-β is tubular epithelial cells which release this cytokine in a paracrine fashion to activate interstitial fibroblasts (45). Clinical and laboratory studies have identified a large number of vitamin D-regulated cellular and tissue processes that potentially suppress renal fibrosis. In 2006, Tan *et* al. showed that paricalcitol treatment was able to suppress the expressions of TGF-β and its respective receptor in a model of obstructive nephropathy (46). Ito et al. also demonstrated that 1,25(OH)_2_D_3_-bound VDR inhibited the TGF-β-SMAD signaling pathway in a mouse model of unilateral ureteral obstruction (UUO) (47). On the other hand, we previously showed that vitamin D deficiency caused a decrease in VDR expression and an increase in TGF-β expression in a model of CKD progression (2). Similarly, in the present study we observed an expected decrease in VDR expression in VDD+Nx rats. In addition to a lower VDR expression, we found an increased expression of TGF-β associated with the highest ratio of FIA in VDD+Nx rats. Thus, our data reinforce the contribution of vitamin D on TGF-β signaling cascade and renal fibrosis formation.

Changes in the quantity and type of ECM in the scarred kidney may affect the cell-matrix interactions that regulate cell function and phenotype (42). This response alters the signals transmitted into the cell from the ECM, which in turn leads to further dedifferentiation of the cells in a vicious cycle that promotes epithelial-mesenchymal transition (EMT) and maintenance of the mesenchymal phenotype (42). We previously demonstrated an increased expression of vimentin and α-SMA, both markers of cellular phenotypic alteration, in the renal cortex of rats under vitamin D deficiency euthanized 60 days after I/R injury (2). In the present study, we observed a higher expression of α-SMA in the renal cortex of VDD+Nx rats, indicating that vitamin D deficiency was implicated in this change. A remarkable study conducted by Xiong *et* al. in a model of UUO demonstrated that loss of VDR is an early event in renal fibrogenesis, considering a potential mechanism coupling renal inflammation and EMT (48). According to the authors, inflammatory cytokines such as tumor necrosis factor α (TNF-α) and interleukin (IL)-1 can suppress the expression of VDR in tubular epithelial cells. Such a loss of VDR could render tubular epithelial cells susceptible to EMT and renal fibrosis induced by TGF-β and depression of β-catenin signaling (48). In our study, we found a reduced expression of VDR and increased expression of TGF-β and α-SMA in VDD+Nx rats, supporting the hypothesis that vitamin D deficiency is not only associated with renal tubulointerstitial damage and fibrosis but also with cellular phenotypic alteration. Therefore, our data emphasize the importance of maintaining adequate levels of vitamin D, specially when a significant kidney injury is already established. Once sufficient, vitamin D could help to restore VDR and retard renal fibrosis formation by blocking EMT.

As discussed earlier, our 5/6 nephrectomy model resulted in exacerbated morphological changes with greater FIA (inflammatory cells infiltrate and fibrosis) mainly in VDD+Nx rats. Macrophages are capable to perform a wide range of critical functions, playing an important role in tissue homeostasis and immune responses in normal and diseased kidneys (19). In fact, by using a broad macrophage marker, we observed an increased expression of CD68+ cells in the renal cortex of VDD+Nx rats, an indicative of a more severe inflammation. Following recruitment to the damaged kidney, macrophages can be broadly classified into two different subtypes: classically activated (M1) and alternatively activated (M2) macrophages (19, 49–51). Commonly, M1 macrophages are considered pro-inflammatory due to their capacity to release cytokines such as IL-1, IL-6, and TNF-α. In contrast, M2 macrophages have a critical role in both anti-inflammatory and tissue repair functions and express arginase, mannose receptor and IL-10, among others (19, 52). Thus, our first set of data concerning CD68+ cells identified that vitamin D deficiency broadly increased the macrophage population as a whole (both M1 and M2 macrophages). Next, we tagged the subpopulation of alternatively activated macrophages (CD206+ cells). By distinguishing the macrophage populations into M1 and M2, we found that vitamin D deficiency not only increased the expression of CD68+ cells (M1+M2 macrophages) but also reduced the expression of CD206+ cells (M2 macrophages). Although M2 macrophages are considered to be pro-fibrotic (53), they also play an important role in tissue repair, especially in acute and active renal lesions. In view of this consideration, we assume that vitamin D deficiency contributed to the extension of the active state of inflammation, maintaining a greater expression of CD68+ cells. Corroborating our data, Zhang et al. suggested that calcitriol treatment inhibited M1 macrophage activation and abated inflammation and podocyte injury in the early phase of experimental type 1 diabetes (DM). In addition, the authors showed that vitamin D enhanced M2 activation in the later stages of DM, conferring protection against renal injury (54). However, how vitamin D regulates macrophage phenotype is not fully understood and further studies are required to elucidate the underlying mechanisms.

Ultimately, we verified that vitamin D deficiency may have impaired the integrity of glomerular vascular endothelium. In our study, we found a lower expression of aminopeptidase P (JG12) in the glomerular capillaries of the VDD+Nx rats. JG12 protein is an aminopeptidase that anchors the cell membrane together with glycosyl-phosphatidyl-inositol and serves as a specific marker for vascular endothelium. Within the glomerular capsule, JG12 is only expressed on the surface of capillary endothelium (55). A damaged renal vascular endothelium is a critical factor involved in the pathological changes present in the progression of CKD. Recently, we demonstrated that vitamin D deficiency impaired renal vascular endothelial function, with the presence of endothelial apoptosis and consequently, a reduction in vascular density (10). Kumar et al. showed that the correction of vitamin D deficiency by cholecalciferol supplementation exerted a positive effect on vascular function in nondiabetic patients with early CKD, likely due to improvement in both endothelial and vascular smooth muscle cell functions (56).

In conclusion, CKD emerges from many pathways that alter the function and structure of the kidney irreversibly. Vitamin D deficiency contributed to the aggravation of functional and hemodynamic renal changes, observed by a greater decline in GFR and higher MAP. Moreover, vitamin D deficiency impaired renal tissue repair probably due to activation of inflammatory pathways, endothelium dysfunction, and fibrosis formation in a moderate-staged CKD experimental model. Thus, our data reinforces the hypothesis that low levels of vitamin D is a risk factor for renal diseases.

## Author contributions

AdB, DC, JG, MS, AS, and RV conceived, designed and performed the experiments. AdB, DC, and RV analyzed the data and contributed to the writing of the manuscript. All authors reviewed the manuscript.

### Conflict of interest statement

The authors declare that the research was conducted in the absence of any commercial or financial relationships that could be construed as a potential conflict of interest.
